# Molecular Dynamics Study of Supramolecular Complexes to the Carbohydrate-Cation System

**DOI:** 10.12688/f1000research.173339.1

**Published:** 2026-04-10

**Authors:** Dhaidan Khalaf kafi, Ali Taher Mohi

**Affiliations:** 1 Medical Physics, University of Fallujah, Fallujah, Iraq, 41001, Iraq; 2 Department of Physics, Mustansiriyah University, Baghdad, Iraq, 10001, Iraq

**Keywords:** molecular dynamic; supramolecular complexes; carbohydrate-cation system; radial distribution function

## Abstract

**Background:**

It is known that calcium ions lead to aggregation of disaccharides, while magnesium ions do not. In this study, simulation results indicated that the cations primarily bind to the sulfate groups of the disaccharides.

**Methods:**

From molecular dynamics simulations, the differences between the calcium and magnesium cation systems can be explained. Additionally, when a modified water model is used in the case of the magnesium system, in which the charges of the water atoms are reduced, the magnesium ions bind the hydration shell less strongly than in the ‘normal’ water model, which facilitates complex formation with the disaccharides. To verify whether the stability differences between the two saccharides are also observed in the simulations, the simulations of the calcium system were repeated with the monosaccharide.

**Results:**

Moreover, result in the formation of carbohydrate-cation-carbohydrate complexes, with the disaccharides primarily binding to the glucose unit, which is attached to the sulfate group. If the cation is not present, the sulfate groups repel each other and complex formation is not possible. The differences between the two cation systems can be explained as follows: Magnesium ions bind much weaker to the disaccharide than calcium ions. The reason is that magnesium ions bind more strongly to the hydration shell due to their smaller ion radius compared to calcium ions. This is energetically more expensive in the case of magnesium ions, which leads to a kinetic hindrance of complex formation compared to the calcium system. The other significant difference between the two cation complexes is that in the case of calcium ions, both disaccharides bind directly to the cation through the sulfate groups.

**Conclusions:**

As expected, the binding energy of the monosaccharide complex was found to be lower than that of the disaccharide complex (6 kJ/mol). The difference in binding energies between the two complexes was relatively small. A significantly lower rupture force was found in the simulations for an individual complex.

## Introduction

Divalent cations frequently play a crucial role in stabilizing supramolecular complexes across various natural systems. These cations act as counterions, facilitating the proximity of negatively charged groups, a key factor in processes such as cell adhesion and aggregation. For instance, cadherins, a group of adhesion proteins, utilize calcium ions to bind together, forming short chains capable of adhering to cell membranes. This mechanism enables the interconnection and networking of multiple cells. ^1, 2^ In other systems, the cations ensure that negatively charged groups can approach and react to one another. For instance, when building DNA, the phosphate group of one nucleotide is linked to the hydroxyl group of another nucleotide. Because these two groups normally repel each other due to negative charges, the reaction is catalyzed by DNA polymerase, which has two magnesium ions in its active center. ^3^ Sea sponge cells self-adhere through a complex that stabilizes through ion pair bonds. This self-adhesion is mediated by aggregation factors, which consist of two proteoglycans. Proteoglycans are macromolecules that consist of a central protein to which one or several glycosaminoglycans are attached. One group of proteoglycans binds to receptors located on the cell surface and ensures that the aggregation factors are bound to the individual cells. The actual adhesion between the cells is achieved by the second class of proteoglycans via calcium ions mediated noncovalent bonding. The bonds of the aggregation factors are very specific, and only bonds between the factors of the same sponge type are observed when the aggregation factors of different sponges are mixed. ^4^ Furthermore, the presence of cations is absolutely necessary for cell adhesion, whereby other metal ions can partially replace calcium in its function. ^5, 6^ The aggregation factors were isolated and identified for various sea sponges, and for the sea sponge Microciona prolifera, the sulfated disaccharide GlcpNAc3S(β1–3) Fucp is relevant for the complex that is stabilized by the calcium ions.

**Figure 1.**
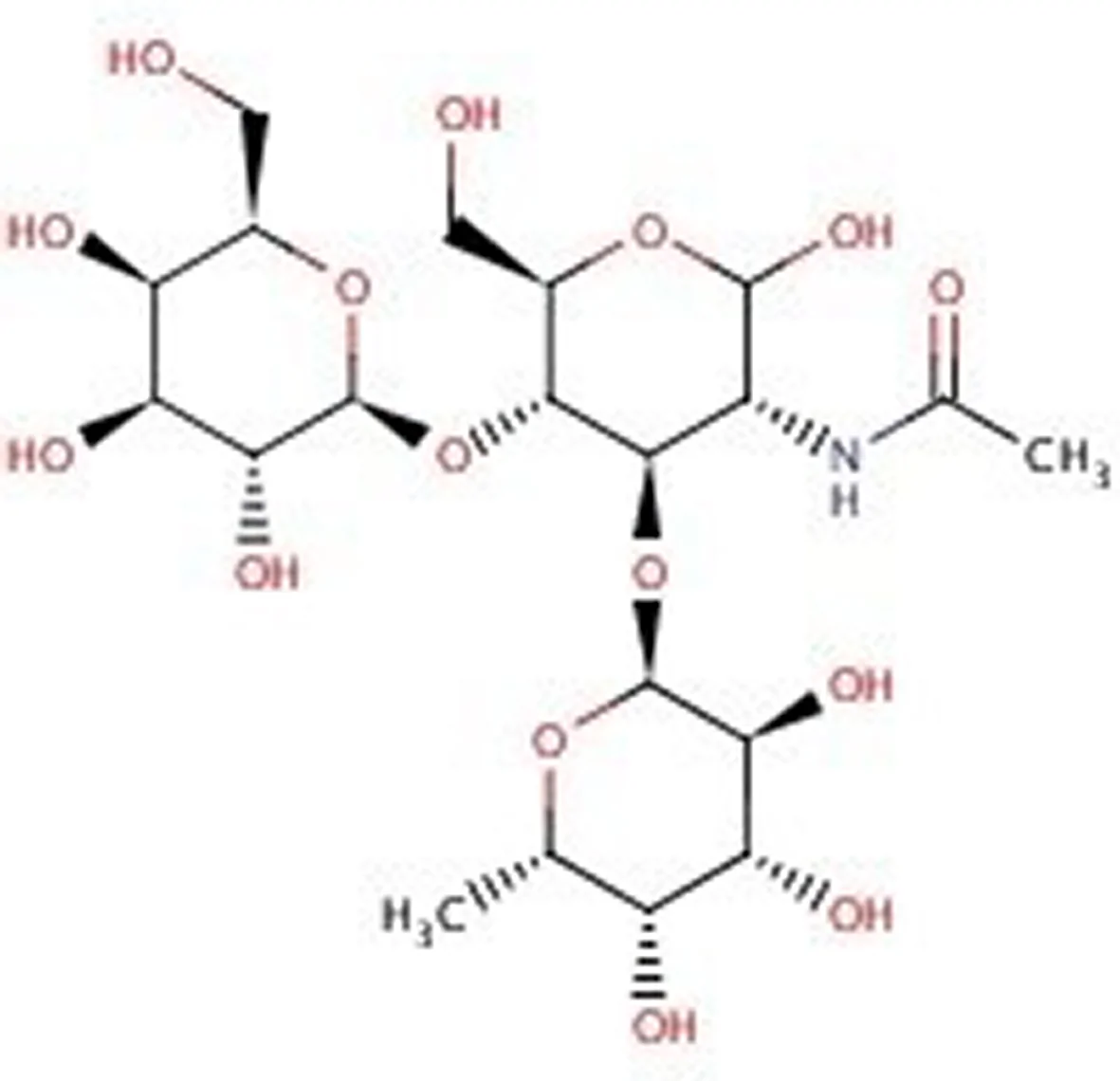
2D and 3D structure of the sulfated disaccharide GlcpNAc3S(β1–3)Fucp.

Fig. 1 presents 2D and 3D structure of the sulfated disaccharide GlcpNAc3S(β1–3) Fucp, which is responsible for the carbohydrate-carbohydrate complex bridged by calcium ions. ‘Glc’ is the abbreviation for a glucose sugar and ‘Fuc’ means a fucose sugar. ‘Ac’ stands for an acetyl group (-CO-CH3) and the curved line indicates the binding to the rest of the proteoglycan. In the simulations, an alkyl chain with 5 carbon chain units was used.

Previously, the aggregation behavior of the disaccharide was investigated as a function of various cations. The results demonstrated that while magnesium ions did not induce aggregation, calcium ions did lead to aggregation. Furthermore, cadmium ions also caused aggregation, although to a lesser extent compared to calcium ions. In a separate study, the disaccharide-cation system (involving the cations Mg2+, Ca2+, and Cd2+) was investigated using atomic force microscopy (AFM). The cantilever tip and surface were coated with the disaccharide. When brought into contact, the sugars and ions had the potential to form complexes. In an aqueous environment without additional cations, repetitive measurements were conducted at small distances (before the tip and surface made direct contact), resulting in a repulsive interaction attributed to the negatively charged disaccharide monolayers. The addition of magnesium ions suppressed this repulsive interaction, but no complex formation was observed. In contrast, binding interactions occurred in 95% of measurements when calcium ions were used, and the rupture force for a single complex was determined to be 30 ± 6 pN. In experiments using cadmium ions, binding events were observed in only about 30% of cases.

Following previous findings, ^7^ the purpose of this study is to investigate the binding mechanism of calcium ion carbohydrate-carbohydrate bond and to determine why no analogous magnesium complex exists. The individual simulations and their results are presented below. All molecular dynamics (MD) simulations were performed using the DFTB+ version 23.1. ^8^ A similar protocol was used as for the simulations of the many different biological systems. ^9, 10^ The 3ob Slater-Koster files were used for modeling the interactions between the atoms. ^11^ The 3ob parameters are optimized for bio and organic molecules. In all simulations, carbohydrates and ions were solvated with water.

In this study, simulations were performed in which multiple disaccharide molecules and cations were modeled in an aqueous medium, with the excess charge of the cations balanced by chlorine ions. Subsequently, different radial distribution functions g(r) were considered to analyze to which extent different groups accumulate in the simulation.

## Simulation methods

The sulfated disaccharide presents a relatively large system, posing a challenge for DFT methods due to the substantial computational demands ^12, 13^ associated with such scale. However, the density functional-based tight-binding method (DFTB) emerges as a rapid and efficient semi-empirical quantum-chemical computational approach. It enables molecular dynamics simulations of systems containing several thousand atoms, with simulation durations in the picosecond range, all without necessitating extensive computational time or significantly augmented computing resources. ^14– 17^ This characteristic renders DFTB a distinctive and highly adaptable tool for exploring not just structure but also dynamics. Our investigations involved conducting molecular dynamics simulations (MD) utilizing the dispersion-corrected density functional-based tight-binding theory (DC-DFTB). ^13^

The following protocol was used in the actual MD simulations. First, the isolated structures were subjected to energy minimization. Subsequently, the system was solvated and water molecules were randomly exchanged with ions. A second energy minimization was performed for the whole system. In the first equilibration phase, the system was subjected to a ‘velocity rescaling’ thermostat 1 with temperature 300 K and a time constant of 0.25 ps for 50 ps. The MD trajectories were generated in the NVT ensemble using the Berendsen thermostat. The simulation box has a size of about 5.4 nm × 4.4 nm × 4.4 nm.

The binding energy of the ions with the system was calculated with MP2 methods using Gaussian simulation package. ^18^ Using the MP2 method to study the full system is very costly in terms of computation. A more feasible and accurate approach is to consider only a part of the system that the MP2 method can handle. This part is called a cluster, and its edges are saturated with hydrogen atoms.

In the preliminary study, a single solvated disaccharide molecule and 20 cations (Ca2+ and Mg2+, respectively) in a cubic simulation box with an edge length of 3.7 nm were modeled for 20 ps. To neutralize the system, 39 chlorine ions were added as counterions.

In simulations with many saccharide molecules (mono- or disaccharide) from which radial distribution functions were obtained, a cubic simulation box with an edge length of 6.5 nm was used, and the system was simulated for 15 ps. Study the full systems with DFTB method in range from nanoseconds to microseconds, with some studies reaching milliseconds for more complex systems, tends to be prohibitively expensive at the current technical level. A compromise between computational cost and accuracy can be achieved by considering only a cluster of the system or educing simulation time. It is assumed that 15 ps is sufficient for observing the specific dynamics of cation-mediated disaccharide aggregation. For many molecular processes, significant conformational changes or aggregation events can occur on the order of picoseconds to nanoseconds, making 165 ps potentially sufficient for capturing initial interactions and dynamics. ^1– 6^

Table 1 shows the number of saccharide molecules, cations (Ca2+ and Mg2+, respectively), and counterions (Cl−) used in individual simulations. In the case of the magnesium system, simulations were repeated with the modified water model. The traditional models for water cannot accurately capture the strength and nature of ion-water interactions, especially in systems with high ionic concentrations or specific ions like calcium and magnesium. This change is aimed at improving the representation of electrostatic interactions, reducing excessive polarization, or better mimicking experimental observations related to hydration properties. This modification lead to improved accuracy in simulating ion solvation and aggregation processes. By adjusting the charge distribution, the model more accurately reflect the dynamics of hydration shells around cations. In the simulations with the modified water model, the charges of the individual atoms of a water molecule were reduced to 83.6% of their original value.

**Table 1.** Number of saccharide molecules, cations (Ca2+ and Mg2+,) and counterions (Cl−) used in the simulations.

saccharides	Cations/Counterions
5	15/25
5	20/35
5	25/45
5	40/75
10	20/30
10	40/70
10	60/110
10	80/150
15	20/25
15	40/65
15	60/105
15	80/145

Three additional simulations were conducted for this simulation box in which the negative charge of disaccharides was balanced by sodium ions, and no other ions were added. These simulations contained 5, 10, and 15 disaccharide molecules and as many sodium ions. These systems were also simulated for 15 ps.

For the calcium system, three more simulations were performed with a larger simulation box (9 nm edge length). Table 2 shows the number of disaccharides, cations (Ca2+), and counterions (Cl-) used in the simulations. The same parameters were used as in the other simulations despite the different number of particles and larger simulation box.

**Table 2.** Number of disaccharide molecules, cations (Ca2+ or Mg2+) and counterions (Cl−), that were used in the simulations with the larger simulation box.

saccharides	Cations/Counterions
30	120/210
30	150/270
30	180/330

## Results and discussion

### Preliminary investigation

Simulations were conducted to study the carbohydrate-cation system, which involved modeling several disaccharide molecules and divalent cations (Ca2+ and Mg2+). ^19^ Before presenting the results of these simulations, a preliminary investigation was carried out to determine to which groups or atoms within the disaccharide magnesium ions bind to. In this investigation, two systems were studied, each containing a solvated disaccharide molecule and 20 cations (Ca2+ or Mg2+). To compensate for the excess positive charge of the cations, 39 chlorine ions were added as counterions1. The simulations showed that the magnesium ions bind to the sulfate group and carbon group of the disaccharide, but not to the acid atoms of the disaccharide, to which the calcium ions have a higher affinity. ^20^ The reason for this is the smaller ionic radius of the magnesium ion compared to the calcium ion, which causes a solvated magnesium ion to bind the water molecules of the hydrate shell to itself more strongly than a calcium ion does. ^21^ The difference between the two ions is also reflected in their hydration enthalpies and the rate constants for the water exchange. ^22^ Table 3 in the source gives the experimentally determined values as well as the cation radius of both types of cations. The cation radius, ^23^ hydration enthalpy ∆Hhydra ^24^ and rate constant for water exchange kex for the two types of cation (Ca2+ and Mg2+). For the Values of kex see the following references: Mg2+ ^25^ and Ca2+. ^26^

**Table 3.** The experimentally determined values as well as the cation radius of both types of cations.

Cation type	Rcation/Å	∆Hhydra/kJ/mol	kex/s − 1
Mg2+	0.60	−1922	5.3 · 105
Ca2+	0.95	−1577	(6–9) · 108

Magnesium has a higher (more negative) hydration enthalpy than calcium, which means that magnesium binds the hydrate shell more strongly than calcium 1 2. This results in slower water exchange in the magnesium system, as the hydrate shell must first be partially broken for the cations to bind to the oxygen atoms of the disaccharide. ^27^ The formation of the cation-disaccharide bond is kinetically inhibited due to the stronger binding of the hydrate shell to magnesium1. To investigate whether the strength of the hydrate shell binding can explain the differences in the affinity of the cation types, further simulations were carried out with magnesium ions1. In these simulations, the water model used was modified by scaling the partial charges of the water molecules by the factor rMg/rCa = 0.84, which reduced the strength of the Coulomb interaction between the magnesium ion and the water molecule1. As a result, the hydrate shell should bind less strongly to the magnesium ion, allowing the cation to attach more readily to the disaccharide. ^28^ The simulations with the modified water model are designated as ‘Mg*’.

### Determination of the RDF

Multiple simulations were conducted to investigate the binding structure and stability of cation-carbohydrate complexes, varying the numbers of disaccharide molecules and cations. For each cation type (Ca, Mg, and Mg*), three disaccharide concentrations with four cation concentrations were examined, resulting in a total of 12 simulations. Table 4 details the specific quantities of disaccharide molecules and cations utilized in the simulations. Chlorine ions were employed to balance the excess charge of the cations in each simulation. Additionally, simulations were performed for the three disaccharide concentrations where the negative charge of the disaccharides was neutralized by sodium ions without the addition of other ions. These simulations included 5, 10, and 15 sodium ions, resulting in only 3 simulations compared to the 12 simulations with other cation types. These sodium ion simulations represented a system without cations, although the presence of sodium ions was necessary for charge balance in the simulation. Experimental evidence suggests that no disaccharide complex forms with sodium ions due to repulsion between deprotonated sulfate groups. Consequently, no complex formation was observed in the MD simulations of the sodium system, serving as a ‘negative blank sample’. The term ‘Na’ was introduced for simulations involving sodium ions. All simulations utilized a cubic box with an edge length of 6.5 nm and were solvated in water. The system was coupled to a thermostat and barostat to regulate temperature (T = 300 K) and pressure (p = 1 bar). Each simulation ran for 15 ps.

**Table 4.** The quantities of disaccharide molecules and cations employed in the simulations.

Disaccharides	cations (Ca, Na, Mg, Mg*)
5	15, 20, 25, 40
10	20, 40, 60, 80
15	20, 40, 60, 80

Table 4 displays the quantities of disaccharide molecules and cations employed in the simulations. Each cation type was studied with three disaccharide concentrations, each paired with four distinct cation concentrations. The quantities of disaccharide molecules and cations for the four cation concentrations are presented in a single line.

### RDF of sugar rings

**Figure 2.**
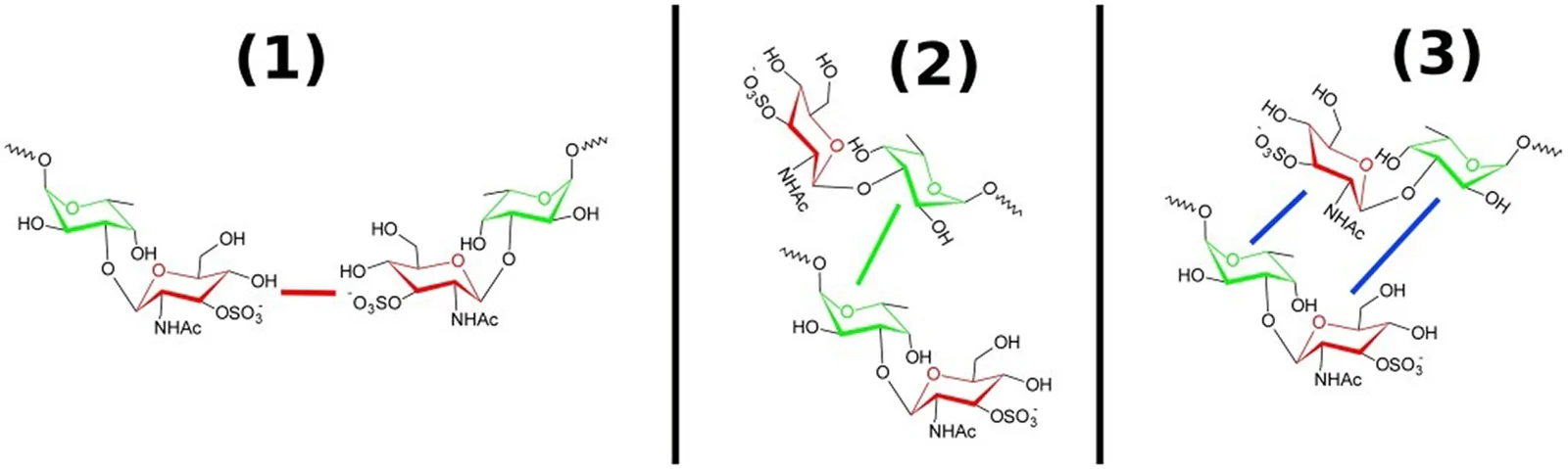
Possible arrangements of disaccharides in the complex. Other similar conformations can also be generated can be produced by rotation of a disaccharide molecule. The colored lines indicate contacts between the same (red or green) and different (blue) sugar rings.

**Figure 3.**
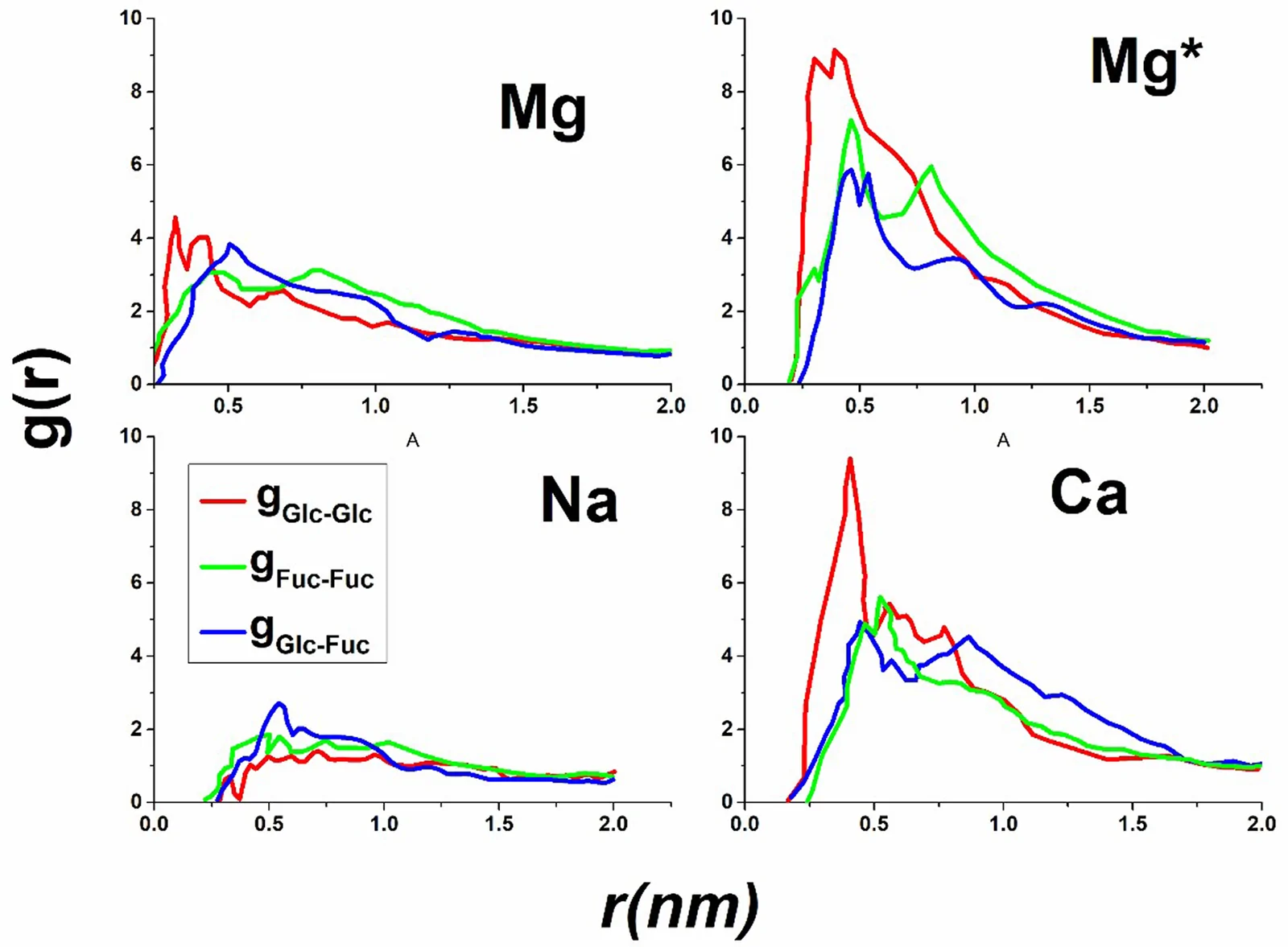
Radial distribution function with respect to the centers of gravity of the sugar rings. The Color scheme is the same as in Figure 2. With the index ‘Glc’, the glucose rings and by ‘Fuc’ meant the fucose rings.

Figure 2 illustrates three potential binding modes for disaccharide molecules to cations. The cations are not depicted, as the focus is on the relative arrangement of disaccharides. In cases (1) and (2), conformations are also considered where one of the two disaccharides is bent out of the plane, resulting in an angled arrangement rather than a linear one. In case 3, the glucose rings to which the sulfate groups are bound are colored red, while the fucose rings to which the alkyl chains are bound are colored green. In cases (1) and (2), where two identical sugar rings bind to a cation, the sugar rings are connected by corresponding colored lines. Interactions between different sugar rings are highlighted in blue, as seen in case (3). For the determination of radial distribution functions, the distances between the centers of the sugar rings (excluding the side groups) were measured. Henceforth, the glucose ring will be referred to with the index ‘Glc’, and the fucose ring with the index ‘Fuc’.By analyzing the three possible radial distribution functions gGlc-Glc, gFuc-Fuc, and gGlc-Fuc, it can be determined whether one of the three possible arrangements (1, 2, or 3) is preferred in the complex. Figure 3 presents the three radial distribution functions for all cation types, using the same color code as in Figure 2. The radial distribution functions were averaged over all simulations for the corresponding cation type. In the calculation of gGlc-Fuc, only the intermolecular contributions to the radial distribution function were considered, as these are the only ones relevant for the subsequent analysis.

Comparing the three radial distribution functions of the sodium system with those of other cation types, two things stand out. On the one hand, the three distribution functions have smaller maximum values (gX-Y < 3) than those of the other cation types (gX-Y > 4). This means that in the case of the sodium system, the disaccharides do not approach each other as much as in the other cation systems. This is especially true for the glucose units, as can be seen from gGlc-Glc (Na) < gFuc-Fuc (Na); gGlc-Fuc (Na) for the sodium system, while the opposite trend is observed for the other cation systems. This trend can be explained by the deprotonated sulfate groups that are bound to the glucose units. The few cations in the sodium system cannot balance the negative charge of the sulfate groups in the same way as the many divalent cations. As a result, no pronounced approach of the disaccharides is observed in the sodium system. It can therefore be assumed that a certain amount of (divalent) cations is essential for the aggregation of the disaccharides.

In the case of calcium ions, a pronounced peak is found in gGlc-Glc (Ca) at about r = 0.6 nm, and it is evident that arrangement (1) from Figure 2 is clearly preferred over the other two arrangements. The divalent cations act here as a kind of “buffer” that balances the negative charge of the sulfate groups and thus enables the approach of these groups. Moving on to magnesium ions (with the normal water model), this pronounced peak is missing in gGlc-Glc (Mg). Nevertheless, gGlc-Glc (Mg) has a larger value for small distances (r ≈ 0.55 nm) than gFuc-Fuc (Mg) and gGlc-Fuc (Mg), which is why a slight preference for arrangement (1) can also be assumed here. The reason for the lack of pronounced peak structure is likely the fact that magnesium ions bind the hydration shell more strongly. As a result, the magnesium ions indirectly bind to the sulfate group via the hydration shell, resulting in a weaker bond than in the calcium system. This leads to the complex being less stable and occurring less frequently over time than in Ca, resulting in gGlc-Glc (Mg) < gGlc-Glc (Ca). When considering the radial distribution functions in the case of the magnesium system with the modified water model gX-Y (Mg*), the relevance of the binding strength of the hydration shell is confirmed. In this system, the hydration shell is bound less strongly than in the magnesium system with the normal water model (Mg). As a result, the magnesium ions can more easily bind to the sulfate group, and especially for small distances, gGlc-Glc (Mg*) > gGlc-Glc (Mg).

When comparing gFuc-Fuc (cation) and gGlc-Fuc (cation), they are quite similar for all types of cations. However, compared to gGlc-Glc (cation), the maxima are found at slightly larger distances. In summary, it can be said that especially for Ca and Mg*, a complex is preferred in which the glucose units bind to each other. However, for such a complex, divalent cations are essential to balance the negative charge of the sulfate groups, which would otherwise repel each other (see gGlc-Glc (Na)).

### RDF of sulfate groups and cations

**Figure 4.**
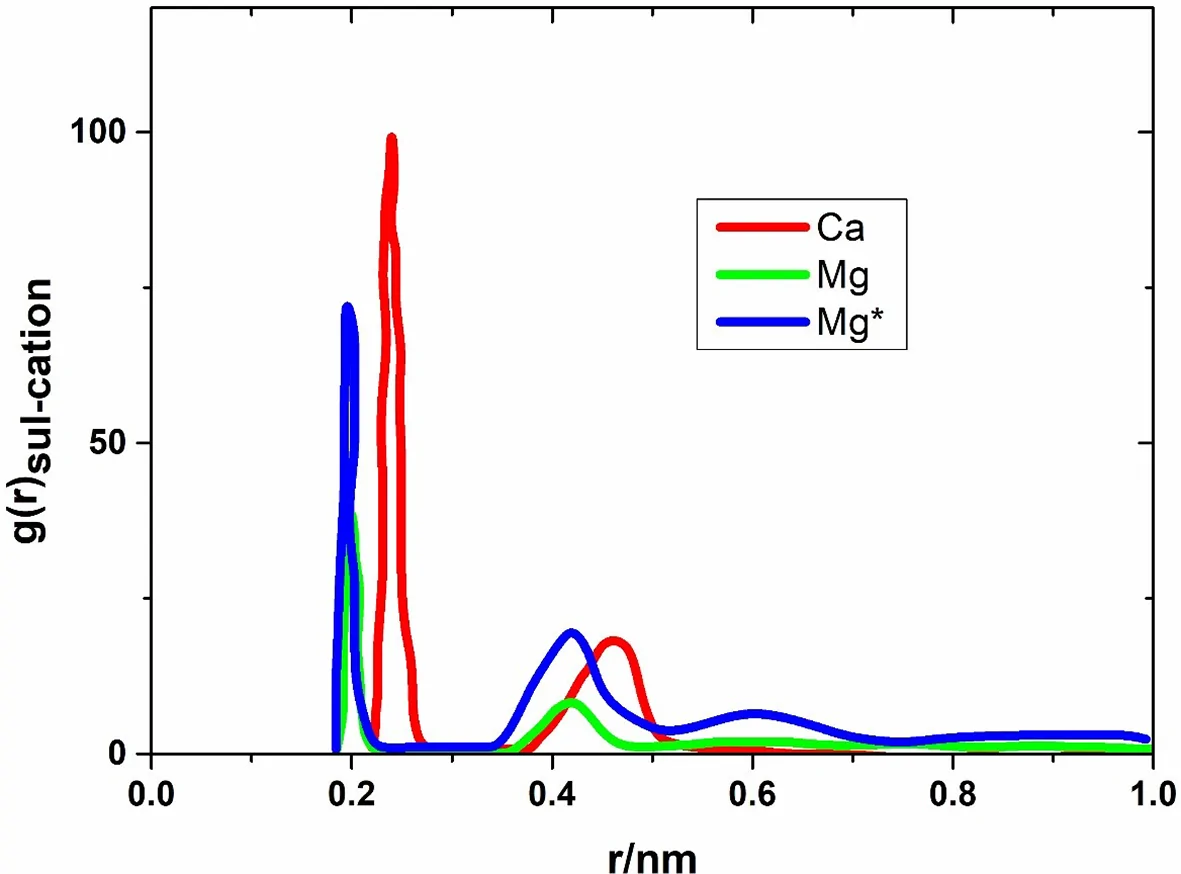
Radial distribution function with respect to the cations and the acidic atoms of the Sulfate groups.

Since the disaccharides primarily bind through the glucose units, it is worthwhile to examine the radial distribution function of the cations and sulfate oxygen atoms, as the sulfate groups are attached to these glucose units. Furthermore, the preliminary investigation discussion noted that the cations predominantly bind to the sulfate groups. Although the carbonyl group of the N-acetyl group is also a preferred binding partner compared to the other oxygen atoms of the disaccharide, it is less preferred than the sulfate group. Before presenting the results, a technical detail should be addressed. When comparing the values of gSul-Kation (rmax), where rmax indicates the position of the first peak, for different cation concentrations at a given disaccharide concentration, it is found that the values decrease with increasing cation concentration. This can be explained by the fact that the number of cations that bind to the sulfate groups does not increase to the same extent as the number of cations modeled in the simulation. This becomes most apparent when considering a single disaccharide molecule. At a certain cation concentration, the sulfate group is saturated with cations. If the cation concentration is increased further, the number of cations that bind to the sulfate group remains unchanged. However, the number of cations that are further away from the sulfate group increases. This leads to an increased probability of not finding a specific cation in the immediate vicinity of the sulfate group, and consequently, the probability of a specific cation binding to the sulfate group decreases. Since the simulations of the different cation types are equally affected by this effect, the results of a given disaccharide and cation concentration can be compared with each other for different cation types. Figure 4 shows the radial distribution function of the sulfate oxygen atoms and cations, gSul-Kation, for the three cation types (Ca, Mg, Mg*), where the results were obtained from simulations with 10 disaccharides and 60 cations. Qualitatively similar results are obtained for the other simulations, and the same trend regarding the affinity of the cation types to bind to the sulfate group is observed.

In the case of calcium ions, gSul-Ca exhibits two peaks at r = 0.24 nm and 0.46 nm. The first peak corresponds to calcium ions that bind directly to the sulfate oxygen atoms. The distance of 0.24 nm is also consistent with the distances found between calcium and oxygen in crystalline sugar-calcium complexes. The second peak corresponds to cations that are indirectly bound to the surrounding sulfate oxygen atoms. This includes calcium ions that bind to the sulfate group via a water molecule. Additionally, this peak is also due to the fact that the sulfate group contains three oxygen atoms to which the cations can bind. If the calcium ion binds to one of the three atoms, it is located near the other two atoms, but the distance is greater. In the case of magnesium ions, the two peaks in the radial distribution function are shifted to smaller distances regardless of the water model used, as smaller binding distances are possible due to the smaller ionic radius of magnesium compared to calcium. In summary, this confirms the assumption that the formation of a bond between the cation and disaccharide in the case of the magnesium system is kinetically hindered due to the more strongly bound hydration shell compared to the calcium system.

### RDF of sulfate groups

The radial distribution function of the sulfate groups is of particular interest for investigating the stability of carbohydrate-cation-carbohydrate complexes. It has already been established that the cations primarily bind to the sulfate group. The results indicate that the disaccharides primarily aggregate through the glucose units to which the sulfate groups are bound. It can therefore be assumed that in the carbohydrate-cation-carbohydrate complex, the disaccharides are bridged by the sulfate groups and cations. Thus, the distance between two sulfate groups is a suitable choice for the reaction coordinate of complex formation or dissociation. Furthermore, it can be ensured that two disaccharides are bridged by a cation when they come very close together, as the groups would repel each other due to their negative charge without the compensating charge of the cation.

The PMF will be presented, as it is essentially a logarithmic representation of the radial distribution function, and therefore, the same information can be obtained from both quantities in principle. However, first, the general trend of gSul-Sul (Ca) for different disaccharide concentrations and different sizes of the simulation box will be briefly discussed. This discussion is more of technical interest; nevertheless, it shows that gSul-Sul (Ca) was adequately determined.

**Figure 5.**
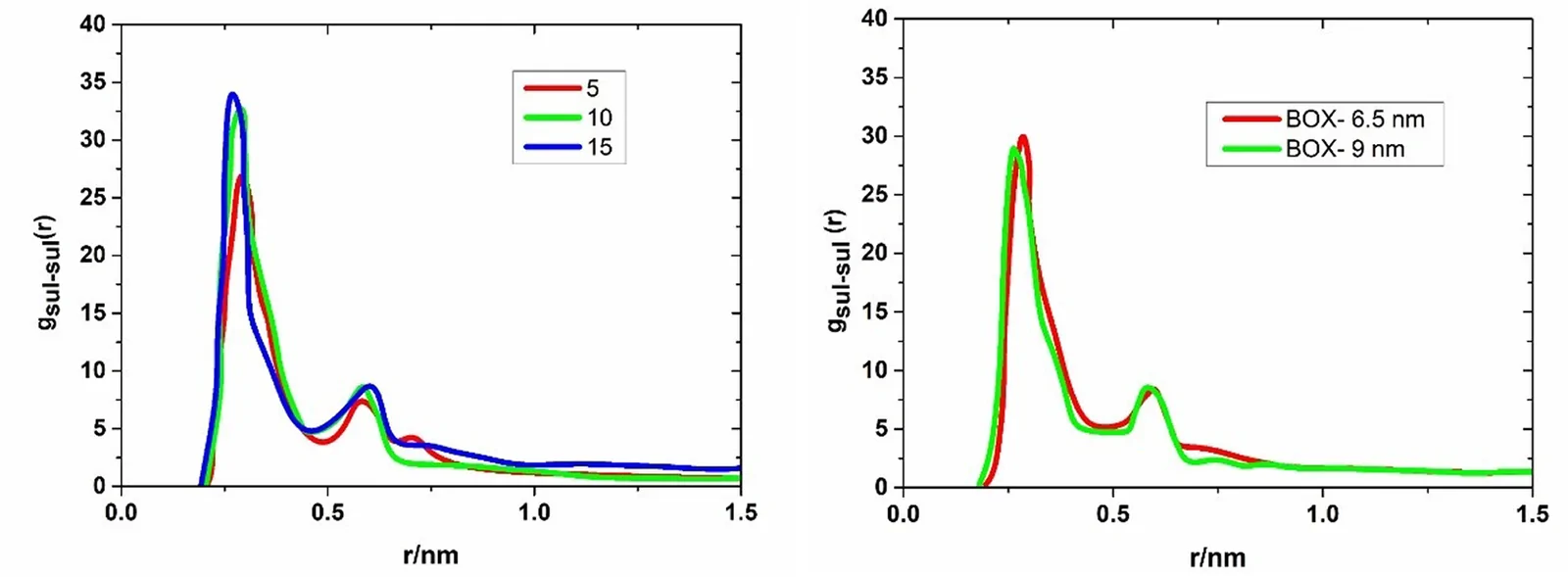
gSul-Sul for the calcium system. Left: gSul-Sul (Ca) at a given disaccharide concentration, where the four calcium concentrations were averaged out. Right: Comparison of gSul-Sul (Ca) for two different simulation boxes, with the smaller box using to determine the radial distribution functions.

When comparing the radial distribution functions of the sulfate groups for different disaccharide and cation concentrations, it is noticeable that they do not always yield exactly the same result, and different values are found. However, unlike gSul-cation, no dependence on the disaccharide or cation concentration can be found. If one averages over the different cation concentrations at a given disaccharide concentration, the results for the three disaccharide concentrations are very similar. This can be seen for the calcium system on the left side of Figure 5. To check whether gSul-Sul (Ca) is affected by the size of the simulation box, further simulations were performed for the calcium system with a larger simulation box (9 nm edge length instead of 6.5 nm). Here, 30 disaccharides and 120, 150, or 180 calcium ions were investigated. On the right side of Figure 5. 6, gSul-Sul (Ca) is shown for the two simulation boxes. In both cases, gSul-Sul (Ca) represents the average of the results for all disaccharide and cation concentrations.

In both figures, it can be seen that the averaged radial distribution functions differ only slightly from each other. Especially since the averaged radial distribution functions for the two different simulation boxes show only small deviations from each other, it can be assumed that gSul-Sul (Ca) was reliably determined, and the same applies to the other cation types. The curves of VCa, VMg, and VMg* have different shapes. VCa has two dips, while VMg and VMg* have only one dip each. To understand why this is the case, it is important to look at the binding structures of each complex. But first, let’s compare VMg* with the other two curves. VMg* is similar to VMg, but it has lower energies overall. This means that the modified water model makes the complex more stable and more frequent than the normal water model. However, VMg* also reaches zero at larger distances than VCa and VMg. This implies that the sulfate groups attract each other more strongly in the modified water model than in the normal water model. This is an unintended effect of using the modified water model, which reduces the partial charges and the dipole moment of water molecules. As a result, the dielectric constant of water decreases and the modified water screens electrostatic interactions less effectively than ‘normal’ water.

### Potential of mean force

A potential of mean force can be calculated from a radial distribution functionusing an equation that describes the free energy along the reaction coordinate r, which is the distance between two sulfate groups. ^29, 30^ The term V cation(r) will be introduced for the PMF of a particular cation system. The PMF can be obtained in Monte Carlo or molecular dynamics simulations to examine how a system’s energy changes as a function of some specific reaction coordinate parameter. It can be a geometrical coordinate or a more general energetic (solvent) coordinate. The PMF is related to the radial distribution function of the system, g(r), by the equation:

$$V_{\mathrm{pmf}}\;\left(r\right)=-K_{B}T\;\ln\;g(r)$$ (1)

where $V_{\mathrm{pmf}}\;\left(r\right)$ is the potential of mean force. The PMF gives the average force over all the configurations of all the particles acting on a particle j at any fixed configuration keeping fixed a set of particles. Potentials of mean force can be calculated from both simulations and experiments run under equilibrium conditions by histogramming the values of the chosen parameter.

**Figure 6.**
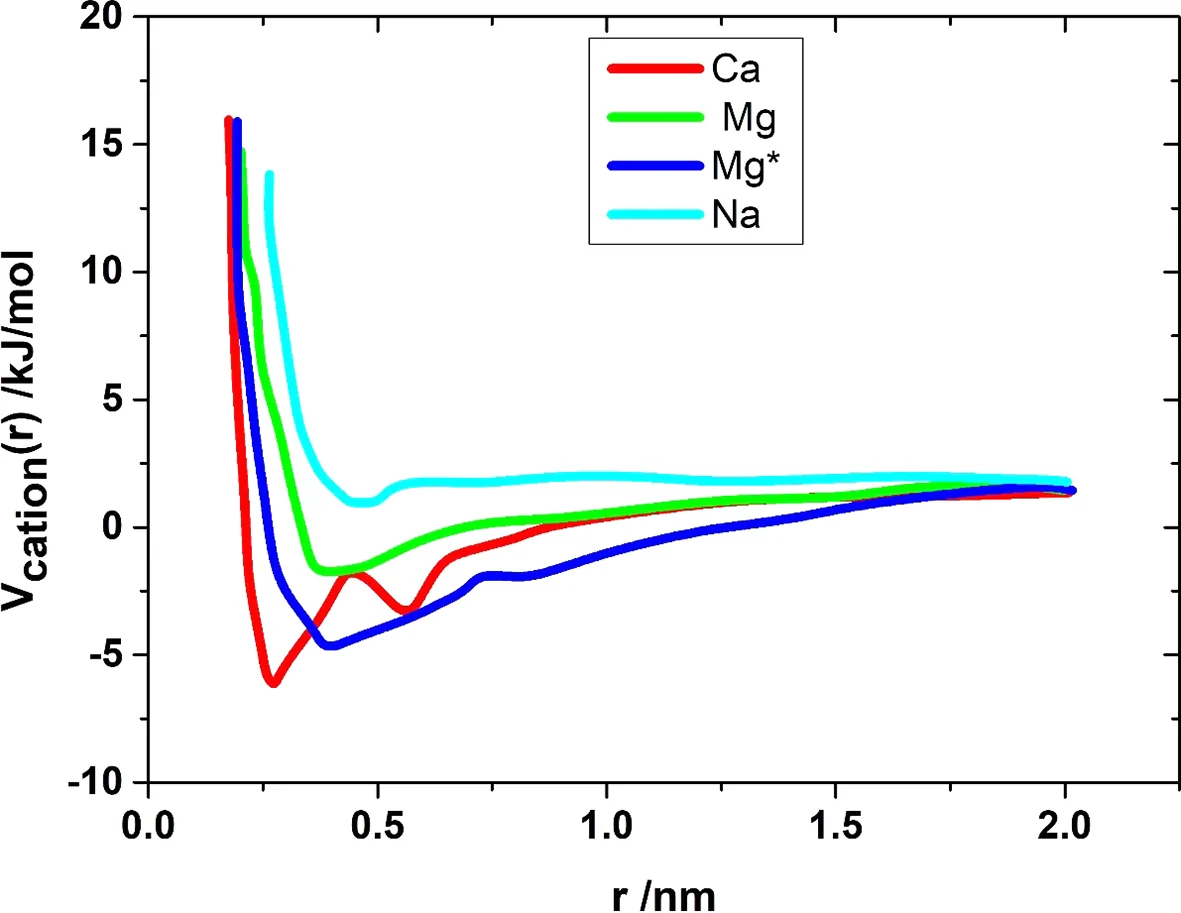
Potential of mean for the carbohydrate-cation-carbohydrate complex, where reaction coordinate, the distance between the sulfate groups is used.

Comparing VCa(r) from Figure 6 with the radial distribution functions from Figure 5, it can be seen that both quantities are equivalent in terms of the essential characteristics of the curve shapes. The maxima of gSul-Sul (Ca) are reflected as minima in VCa(r), and both curves have a constant value from r ≈ 1 nm onwards.

When considering the PMF of the sodium system VNa(r), it is noticeable that it practically does not have a minimum compared to the other cation systems and already increases strongly at larger distances. Since these simulations only contain a few monovalent cations, the curve can be explained by the electrostatic repulsion of the sulfate groups. In the three other systems, the divalent cations can compensate for the negative charge of the sulfate groups, and as a result, the sulfate groups can approach each other further. This can be seen from the fact that the repulsive range of VKation(r) for Ca, Mg, and Mg* is shifted to smaller distances compared to VNa(r). Furthermore, negative values are observed in VKation(r) for the divalent cations, meaning that the sulfate groups experience an attractive interaction at these distances. The distances at which the minima are found correspond to the distance between the two sulfate groups in the bound complex. The minimum of the PMF corresponds to the negative binding energy. For the three systems of divalent cations, the binding energies are found in descending order: 8.5 kJ/mol (Ca), 6.9 kJ/mol (Mg*), and 3.9 kJ/mol (Mg) as Table 5.

**Table 5.** Quantitative summary of binding energies and characteristic distances from RDF and PMF simulations.

System	Binding Energy (kJ/mol)	g_Sul-Sul Peak Position (nm)	g_Sul-Cation 1st Peak (nm)
Ca ^2+^	−8.5	~0.6	0.24
Mg ^2+^	−3.9	~0.55	0.22
Mg*	−6.9	~0.54	0.21

The trend of the binding energies is similar to the trend of the affinity of the cation types to bind to the sulfate group. To estimate an experimental value for the lower bound of the binding energy, the results of pull experiments are suitable. In reference, the mean rupture force of a single calcium complex was determined to be F ≈ 30 ± 6 pN. The distance between the bound state and the transition state was estimated in reference ^31^ to be ∆x ≈ 0.3 nm. The product of these two values corresponds to the amount by which the energy barrier is lowered on average before the transition occurs. Since the transition generally occurs through thermal activation, the energy barrier and thus the binding energy are greater than the amount of the lowering. For the lowering of the energy barrier, one obtains ∆V ≈ F · ∆x = 5.4 kJ/mol, and it can be assumed that the error is about 3 kJ/mol. The value of this lower bound is at least of the same order of magnitude as the binding energy determined here, but it cannot be said by how much the actual value is greater. To achieve better comparability with the experiments, pull experiments were simulated using Brownian simulations. The rupture force that can be determined from these can be compared with the experimental rupture forces and thus provides a better comparison value than the binding energies.

In all three cases, the binding energy is in the order of magnitude of the thermal energy kBT ≈ 2.5 kJ/mol (for T = 300 K). The difference in binding energies between the calcium and magnesium complexes can probably explain why the latter is not observed in experiments. For the magnesium complex, the binding energy is not even twice the thermal energy, meaning that the complex should be easily opened by thermal fluctuations. Thermal fluctuations as the variations in energy that occur due to thermal motion at a given temperature. These fluctuations can affect molecular interactions and stability, making it important to consider them when analyzing complex formation. If thermal fluctuations are large relative to binding energies, this could lead to transient disruptions in complex formation, affecting overall stability and aggregation behavior. In all three cases, the binding energy is in the order of magnitude of the thermal energy kBT ≈ 2:5 kJ/mol (for T = 300 K). The difference in the binding energies for the calcium and magnesium complex can probably explain why the latter is not observed in the experiments. For the magnesium complex, the binding energy is not even twice the thermal energy, which means that the complex should be able to be opened very easily by thermal fluctuations. In the case of the calcium complex, however, the binding energy is more than a factor of 3 greater than the thermal energy, which is why this complex should be somewhat more stable than the magnesium complex. Since the binding energy is nevertheless quite small, it is also to be expected that the calcium complex can dissociate even through thermal fluctuations. However, a single complex is negligible for cell aggregation, and only many such complex bonds bind the cells together.

In the case of the calcium complex, however, the binding energy is more than a factor of 3 greater than the thermal energy, so this complex should be slightly more stable than the magnesium complex. However, since the binding energy is still quite small, it can be expected that the calcium complex can also dissociate due to thermal fluctuations. For cell aggregation, however, a single complex can be neglected, and it is only many such “complex bindings” that bind the sponge cells together.

### Comparison with a monosaccharide system

The previous results show that sulfate groups, which are bound to the glucose unit of the disaccharide, are mainly responsible for complex formation. Experimentally, it was shown in ^32, 33^ that a monosaccharide consisting only of the glucose unit of the disaccharide presented here does not form a stable carbohydrate-calcium-carbohydrate complex. In the experiments, gold nanoparticles were coated with the disaccharide or monosaccharide. When calcium ions were added to a solution of such nanoparticles, aggregation of the nanoparticles was observed in the case of the disaccharide system. Since this aggregation was absent in the case of the monosaccharide system, it was concluded that no carbohydrate-calcium-carbohydrate complex is formed with the monosaccharide.

**Figure 7.**
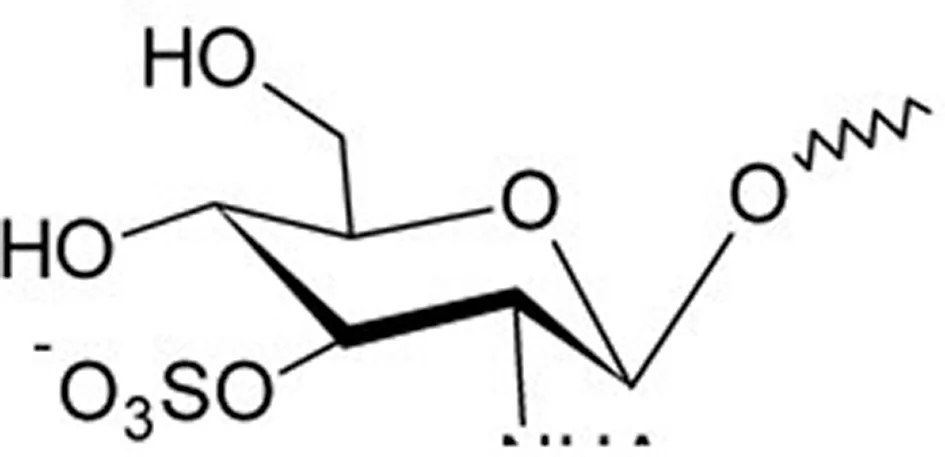
Monosaccharide that consists only of the glucose unit of the disaccharide.

In MD simulations, differences in complex formation between the two saccharides should also be detectable. Therefore, the same simulations as for the disaccharide system were performed with the monosaccharide shown in Figure 7.

**Figure 8.**
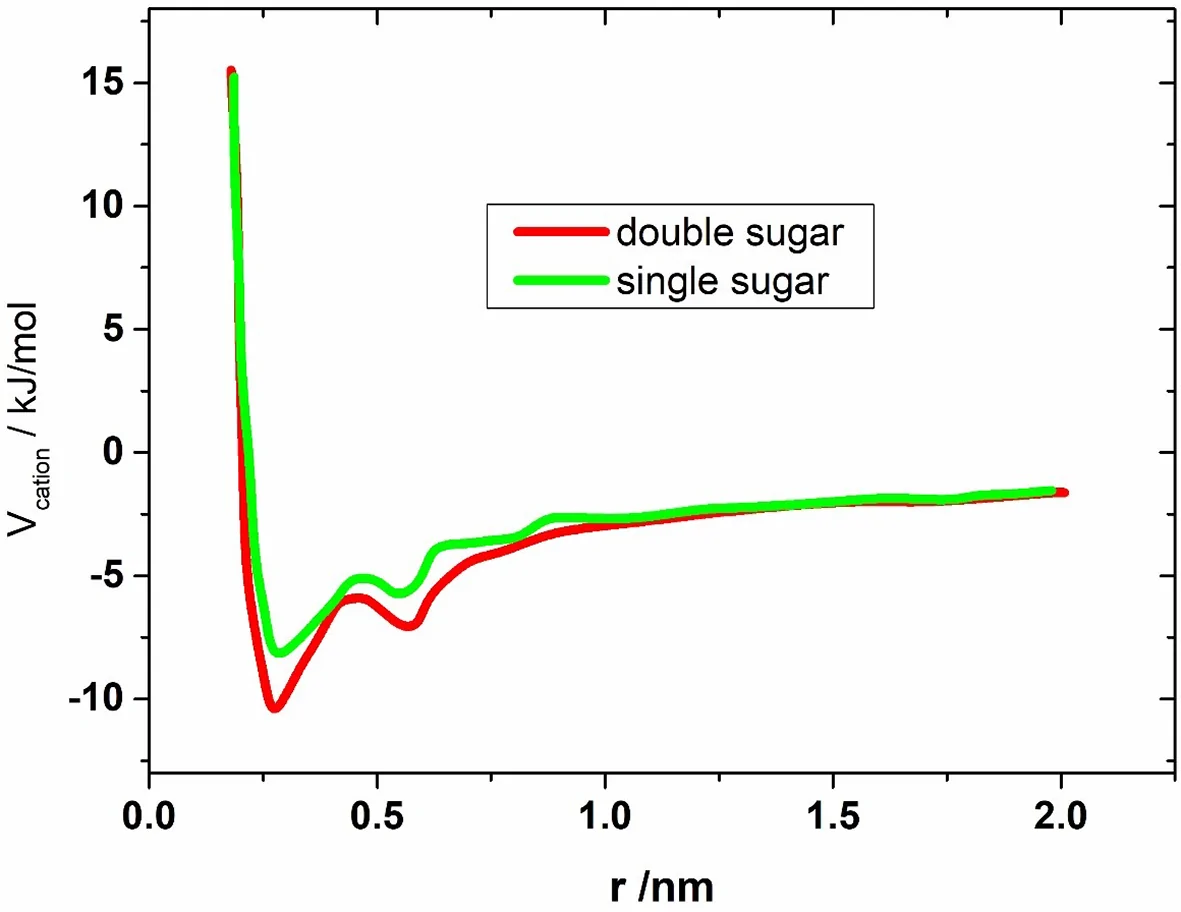
Potential of mean force for the carbohydrate-calcium-carbohydrate complex for the disaccharide and monosaccharide system.

Figure 8 shows the PMF in the case of calcium ions for the two saccharide systems. Ignoring the numerical values of Vcation(r), the course of the PMF for both systems is the same. This is expected because the immediate environment of the sulfate groups, whose distance serves as a reaction coordinate, is the same in both systems. However, in the case of the monosaccharide, a smaller binding energy is observed (≈ 6 kJ/mol instead of 8.5 kJ/mol). This is thus one unit of thermal energy kBT smaller than for the disaccharide system. The fact that the monosaccharide system is less stable than the disaccharide system is positive in terms of comparability with the experiments. Nevertheless, the relatively small energy difference is striking.

Finally, it is worth mentioning that trajectory lengths of DFTB simulations of 15 ps may not potentially enough for the studied system. However, one may emphasize that only preliminary analyses have been conducted in this study (e.g., energy convergence and structural stability), which suggest that the system reaches a near-equilibrium state within this time frame. Longer simulations could provide further insights into dynamic behavior, hence exploring options for extending simulations in future work is demanded.

## Conclusion

This research investigates the cation-mediated aggregation of disaccharides, which is relevant for the self-adhesion of cells in the sea sponge Microciona prolifera. Experimentally, it is known that calcium ions induce aggregation of disaccharides, while magnesium ions do not. The goal of this study was to determine the differences in binding behavior between the two cation species. Initial findings revealed that the cations primarily bind to the sulfate groups of the disaccharides, leading to the formation of carbohydrate-cation-carbohydrate complexes. In these complexes, the disaccharides predominantly bind to the glucose unit attached to the sulfate group. This binding preference is particularly pronounced in the calcium system. To assess the stability of the complexes, a potential of mean force was determined using the distance between the sulfate groups as the reaction coordinate, as small distances between these groups indicate that they are bridged by a cation. In the absence of cations, the sulfate groups repel each other, preventing complex formation. The binding energy for the calcium complex was found to be 8.5 kJ/mol, while in the case of the magnesium complex, it is 3.9 kJ/mol. In both cases, the binding energy is relatively small, consistent with experimental observations that the calcium complex is not very stable and the magnesium complex is not observed. The binding energies are significantly higher in the presence of certain cations (like Ca ^2+^ or Mg ^2+^), this could indicate that these ions play a critical role in stabilizing disaccharide interactions.

MD simulations provide insights into the differences between the two cation systems: Magnesium ions bind much more weakly to the disaccharide compared to calcium ions. This can be attributed to the smaller ionic radius of magnesium ions, which results in stronger binding to the hydration shell. For a cation to bind to the disaccharide, the hydration shell must first be broken, which is energetically more costly for magnesium ions, leading to a kinetic hindrance of complex formation relative to the calcium system. When a modified water model is used for the magnesium system, where the charges of the water atoms are reduced, the magnesium ions exhibit weaker binding to the hydration shell, facilitating complex formation with the disaccharides. Another significant difference is that in the calcium system, both disaccharides bind directly to the cation through the sulfate groups, while in the magnesium system, only one disaccharide enters a direct binding, with the other binding indirectly through a water molecule, weakening the overall binding strength. The results indicate that the sulfate group is crucial for complex formation. Experimentally, it is known that a monosaccharide, consisting only of the glucose unit of the disaccharide, does not form a stable carbohydrate-calcium complex when bound to the sulfate group. To verify whether the stability differences between the two saccharides are also observed in the simulations, the calcium system simulations were repeated with a monosaccharide. As expected, the binding energy of the monosaccharide complex was found to be lower than that of the disaccharide complex (6 kJ/mol), but the difference in binding energies between the two complexes was relatively small. Additionally, adhesion clusters of different sizes stabilized through multiple complex bindings were modeled. These simulations allowed for the determination of the average rupture force of an individual complex. Compared to experimental data, the simulations predicted a significantly lower rupture force for an individual complex, which can be attributed to simplifications in the models used.

Moreover, RDF simulation results showed that the cations primarily bind to the sulfate group. it was shown that the disaccharides primarily aggregate through the glucose units to which the sulfate groups are bound. It can therefore be assumed that in the carbohydrate-cation-carbohydrate complex, the disaccharides are bridged by the sulfate groups and cations. Thus, the distance between two sulfate groups is a suitable choice for the reaction coordinate of complex formation or dissociation. Furthermore, it can be ensured that two disaccharides are bridged by a cation when they come very close together, as the groups would repel each other due to their negative charge without the compensating charge of the cation.

Nevertheless, it is remarkable that the simulations qualitatively reproduced the experiments, as differences in the stability of the complexes formed by the different saccharide-cation systems were observed, this study provides a multiscale understanding bridging molecular physics and biological function.

Preprint:

This work is based on our previously preprint, where large sections of this manuscript have appeared on a preprint server, DOI: 10.21203/rs.3.rs-4630635/v1, Posted Date: July 19th, 2024. This study did not involve human participants, human data, or human tissue.

## Data Availability

All data generated or analyzed during this study are fully represented in the figures and results presented within this article. No separate underlying numerical datasets exist beyond what is shown in the graphs and tables provided.
