# Supplementary material for: Molecular Dynamics Study of Supramolecular Complexes to the Carbohydrate-Cation System
Source: F1000Res. 2026 Aug 21;15:503. Originally published 2026 Apr 10. [Version 2] doi: 10.12688/f1000research.173339.2 (PMC13571022; doi:10.12688/f1000research.173339.2)
Supplement: Peer review history

# Reviewer report: Reviewer response for version 2

**DOI:** 10.5256/f1000research.204795.r513444

**Published:** 2026-09-12

**Authors:** Thuat T. Trinh

**Affiliations:** 1 Norwegian University of Science and Technology, Trondheim, Norway

The authors have responded well to the feedback, leading to a significantly improved manuscript. I recommend indexing.

Is the work clearly and accurately presented and does it cite the current literature?

Partly

If applicable, is the statistical analysis and its interpretation appropriate?

Partly

Are all the source data underlying the results available to ensure full reproducibility?

Partly

Is the study design appropriate and is the work technically sound?

Partly

Are the conclusions drawn adequately supported by the results?

No

Are sufficient details of methods and analysis provided to allow replication by others?

Yes

Reviewer Expertise:

NA

I confirm that I have read this submission and believe that I have an appropriate level of expertise to confirm that it is of an acceptable scientific standard.

# Reviewer report: Reviewer response for version 1

**DOI:** 10.5256/f1000research.191145.r481856

**Published:** 2026-06-03

**Authors:** Weiwei He

**Affiliations:** 1 Chemistry, New York University Abu Dhabi (Ringgold ID: 167632), Abu Dhabi, Abu Dhabi, United Arab Emirates

**1. Typographical and formatting issues should be corrected throughout the manuscript.**

 The manuscript contains multiple typographical, formatting, and citation errors that affect readability. For example, the phrase “magnesium binds the hydrate shell more strongly than calcium 1 2” appears to contain broken citation formatting. The authors should carefully check the entire manuscript, correct all citation-formatting errors, remove duplicated text, and ensure consistent use of chemical terms, symbols, units, and figure labels.

**2. The artificial modification of the Mg²⁺–water interaction is not sufficiently justified.**

 A central part of the manuscript relies on a modified Mg system, in which water charges are scaled to weaken the Mg²⁺ hydration shell. This modification is highly artificial, because changing water charges affects not only Mg²⁺ hydration, but also the water dipole moment, dielectric screening, hydrogen bonding, ion–ion interactions, and sulfate–sulfate electrostatics.

 The Mg* model therefore cannot be used as direct evidence for the proposed mechanism without systematic parameter refinement, benchmarking, and physical validation. The authors are encouraged to perform additional simulations using existing, validated Mg²⁺, Ca²⁺, and Na⁺ ion parameters developed for classical biomolecular force fields, especially those tested for nucleic acid or phosphate/sulfate-rich systems. Relevant examples include DOI: 10.1021/jz201501a, 10.1021/acs.jctc.3c00520, and 10.1002/bip.22868. Such validation is needed to determine whether the reported Ca²⁺/Mg²⁺ selectivity is robust or model-dependent.

**3. The computational model and simulation protocol require substantial clarification.**

 The manuscript states that the MD simulations were performed using DFTB+, but the actual molecular model is not sufficiently described. The authors should clarify whether the simulations are fully DFTB-based, classical MD, or a hybrid workflow involving DFTB-MD and MP2 cluster calculations.

 More details are needed on how water, ions, saccharides, and sulfate groups were modeled. For example, what water description was used, and how are the Mg²⁺, Ca²⁺, Na⁺, and Cl⁻ ions treated in the DFTB framework? The authors should also provide the relevant parameters, timestep, convergence criteria, etc. Without these details, the simulations are difficult to reproduce or critically assess.

**4. The simulation time is too short to support reliable RDF, PMF, and aggregation conclusions.**

 The main RDF and PMF analyses appear to be based on very short simulations, approximately 15 ps for the multi-saccharide systems. This is likely insufficient for sampling divalent-ion dehydration, sulfate coordination, carbohydrate reorientation, solvent relaxation, and supramolecular aggregation.

**5. The PMF-derived binding energies require stronger justification and uncertainty estimates.**

 The manuscript derives PMFs directly from RDFs and interprets the resulting minima as binding energies. This treatment requires much stronger justification, especially because the reported binding energies are small and comparable to only a few kBT.

 The authors should provide convergence analysis and uncertainty estimates for the RDF-derived PMFs. They should also clarify that an RDF-derived PMF in a multi-component, finite-concentration system is an effective potential rather than a rigorous standard binding free energy. To obtain quantitative binding free energies, enhanced-sampling methods such as umbrella sampling or metadynamics are recommended, ideally using reaction coordinates that distinguish direct cation bridging from water-mediated or non-bridged contacts.

Is the work clearly and accurately presented and does it cite the current literature?

Partly

If applicable, is the statistical analysis and its interpretation appropriate?

No

Are all the source data underlying the results available to ensure full reproducibility?

No

Is the study design appropriate and is the work technically sound?

Partly

Are the conclusions drawn adequately supported by the results?

Partly

Are sufficient details of methods and analysis provided to allow replication by others?

No

Reviewer Expertise:

Computational chemistry, force fields, MD simulations

I confirm that I have read this submission and believe that I have an appropriate level of expertise to confirm that it is of an acceptable scientific standard, however I have significant reservations, as outlined above.

# Reviewer report: Reviewer response for version 1

**DOI:** 10.5256/f1000research.191145.r484822

**Published:** 2026-06-02

**Authors:** Lei Dong

**Affiliations:** 1 Ocean University of China, Qingdao, China

The author presents a molecular dynamics simulation study focusing on the supramolecular complexes of carbohydrate-cation systems. This research aims to elucidate the microscopic mechanisms underlying the cation-mediated aggregation of disaccharides, specifically exploring why calcium ions (Ca ^2+^) induce stable disaccharide aggregation while magnesium ions (Mg ^2+^) do not. The author proposes a mechanistic framework based on the kinetic hindrance of the ion hydration shell and direct/indirect coordination with sulfate groups. While the manuscript presents interesting findings, major revisions are required to address the following concerns:

1. There is a critical error in the chemical structure presentation in Figure 1. Despite the heavy emphasis throughout the manuscript on the sulfate group as the core binding site, no sulfate groups are depicted in the chemical structures of the disaccharides in Figure 1. Furthermore, while the figure caption claims to display both 2D and 3D structures, only 2D skeletal formulas are visible in the figure. The author must completely redraw Figure 1 to ensure technical accuracy and consistency between the text and the illustration.
2. The simulation timescale is severely insufficient. The production run lasted for only 15 ps, which is far too short to achieve thermodynamic equilibrium for flexible carbohydrate chains characterized by rich conformational degrees of freedom. Such limited sampling compromises the reliability and accuracy of the resulting radial distribution functions (RDF) and potentials of mean force (PMF). The author is strongly encouraged to extend the simulation timescale to the nanosecond or microsecond range.
3. The physical validity of the modified water model is highly questionable. To artificially weaken the hydration shell of magnesium ions, the author scaled down the atomic charges of water to 83.6% (referred to as the Mg* model). This ad hoc modification alters the intrinsic physical properties of bulk water, such as its dipole moment and dielectric constant. The author must provide comprehensive benchmark data evaluating how this modification impacts the fundamental physical properties of water.
4. There is a logical disconnection between the simulated binding energies and the observed macroscopic phenomena. The simulation indicates that the monosaccharide complex still possesses a binding energy of approximately 6kJ/mo, which is significantly higher than the environmental thermal fluctuation background ( 2.5 kJ/mol); thus, complex formation should theoretically be observable. However, the experimental control showed that monosaccharide-functionalized gold nanoparticles do not undergo any aggregation. The author needs to provide a thorough and rigorous explanation for this quantitative contradiction.
5. The manuscript suffers from poor text preparation and severe text duplication. For instance, on Page 11, a long paragraph discussing the binding energy being in the same order of magnitude as thermal energy (" *In the case of the calcium complex….*") is repeated word-for-word in the consecutive text. Additionally, the structure of the conclusions section appears disorganized. A meticulous proofreading, language polishing, and structural refinement are mandatory for the entire manuscript.

Is the work clearly and accurately presented and does it cite the current literature?

Partly

If applicable, is the statistical analysis and its interpretation appropriate?

Partly

Are all the source data underlying the results available to ensure full reproducibility?

Partly

Is the study design appropriate and is the work technically sound?

Partly

Are the conclusions drawn adequately supported by the results?

No

Are sufficient details of methods and analysis provided to allow replication by others?

Yes

Reviewer Expertise:

Controlled Synthesis and Applications of Inorganic Nanomaterials；Synthesis of Acrylic Polymers and Their Applications in Coatings；Development of Environmentally Friendly Marine Protective Coatings

I confirm that I have read this submission and believe that I have an appropriate level of expertise to confirm that it is of an acceptable scientific standard, however I have significant reservations, as outlined above.

# Reviewer report: Reviewer response for version 1

**DOI:** 10.5256/f1000research.191145.r481858

**Published:** 2026-05-27

**Authors:** Thuat T. Trinh

**Affiliations:** 1 Norwegian University of Science and Technology, Trondheim, Norway

This manuscript presents a molecular dynamics (MD) study investigating the binding mechanisms of cation-mediated carbohydrate-carbohydrate complex formation, specifically examining the differences between calcium (Ca²⁺) and magnesium (Mg²⁺) ions in facilitating aggregation of sulfated disaccharides. The work builds upon experimental observations that calcium ions induce aggregation while magnesium ions do not, and aims to elucidate the molecular-level mechanisms underlying this behavior. The authors employ density functional tight-binding (DFTB) simulations combined with radial distribution function (RDF) and potential of mean force (PMF) analyses to investigate cation-carbohydrate interactions. The study explores various simulation conditions including different cation concentrations, system sizes, and a modified water model for magnesium to test the hydration shell hypothesis.

 The manuscript provides a systematic investigation of cation-carbohydrate interactions with reasonable coverage of relevant parameters. However, significant concerns exist regarding the simulation methodology, timescale sufficiency, and rigor of the computational approach that require careful consideration.

 Comments:

 1. The most significant concern regarding the computational methodology is the extremely short simulation duration of 15 ps for production runs. This time scale is orders of magnitude shorter than typical production MD simulations for biomolecular systems. While DFTB is more computationally demanding than classical force field MD, 15 ps is insufficient to adequately sample conformational space, especially for:

 - Cation binding/unbinding events typically require microseconds to milliseconds of sampling for proper convergence.

  - While RDFs can converge relatively quickly, the statistical uncertainties are likely large given the short sampling. Block averaging over multiple independent trajectories is essential.

 - The carbohydrate-cation-carbohydrate binding energies reported (3.9-8.5 kJ/mol) are small and thermal fluctuations are significant. Longer simulations with independent replicas are needed to establish reliable binding free energy estimates.

 The authors should  extend the simulation time to at least 100-500 ps and run multiple independent replicas, 

 2. The use of a modified water model for magnesium simulations, where atomic partial charges are scaled to 83.6% of their original values, is a significant methodological choice that requires substantial justification and validation. 

 This approach raises several concerns:

 -Scaling water charges arbitrarily modifies fundamental electrostatic properties including the dipole moment and dielectric constant. What is the theoretical justification for choosing the factor of 0.83 (rMg/rCa)?

 -The authors should validate the modified water model against experimental properties of water and aqueous Mg²⁺ solutions (density, dielectric constant, hydration structure, ion-water binding energies) to demonstrate that it provides improved representation compared to the standard model.

 -The modified model likely alters hydrogen bonding networks, proton transfer rates, and other water-mediated processes. The authors acknowledge (line 224) that this may cause unintended effects including reduced dielectric screening. A more rigorous approach would be to systematically study how water model selection affects the key observables.

 Without this validation, conclusions drawn from the modified water model simulations must be treated with caution, as they may reflect artifacts of the ad hoc parameter modification rather than genuine physical effects.

 3. For a reliable PMF calculation from RDFs, the RDF must be well-converged across all reaction coordinate values. With only 15 ps of simulation time and no independent replicas or block averaging reported, the statistical uncertainties are unknown and likely large.

 - Binding energies of 3.9-8.5 kJ/mol are reported with no error bars or confidence intervals. Given the small magnitude relative to thermal energy (kBT ≈ 2.5 kJ/mol), statistical significance cannot be established without error analysis.

 - More robust approaches for calculating binding free energies exist, including umbrella sampling, thermodynamic integration, or metadynamics. While these methods are more computationally demanding, they would provide more reliable quantitative estimates.

 4. The manuscript compares the simulated binding energies (ΔV ≈ 5.4 kJ/mol lower bound from experimental rupture force) with experimental AFM pull experiments measuring rupture forces from reference 31 (Lorenz et al. 2012).While this comparison is useful, several issues require clarification:

 - Rupture force measurements probe kinetic properties (energy barriers) while PMF calculations yield thermodynamic free energies. The relationship between these quantities is complex and depends on the pulling rate, cantilever spring constant, and molecular dynamics of the dissociation pathway.

 - The treatment assumes a simple Bell model with a single energy barrier, which may not capture the more complex energy landscape of carbohydrate-cation-carbohydrate interactions.

Is the work clearly and accurately presented and does it cite the current literature?

Partly

If applicable, is the statistical analysis and its interpretation appropriate?

Partly

Are all the source data underlying the results available to ensure full reproducibility?

Partly

Is the study design appropriate and is the work technically sound?

Partly

Are the conclusions drawn adequately supported by the results?

No

Are sufficient details of methods and analysis provided to allow replication by others?

Yes

Reviewer Expertise:

Atomistic simulations

I confirm that I have read this submission and believe that I have an appropriate level of expertise to confirm that it is of an acceptable scientific standard, however I have significant reservations, as outlined above.

# Response

**Published:** 2026-06-23

**Authors:** Dhaidan Khalaf kafi

**Affiliations:** Medical Physics, University of Fallujah, Al-Fallujah, Al Anbar Governorate, Iraq

**1. **The most significant concern regarding the computational methodology is the extremely short simulation duration of 15 ps for production runs. This time scale is orders of magnitude shorter than typical production MD simulations for biomolecular systems. While DFTB is more computationally demanding than classical force field MD, 15 ps is insufficient to adequately sample conformational space, especially for:

 - Cation binding/unbinding events typically require microseconds to milliseconds of sampling for proper convergence.

  - While RDFs can converge relatively quickly, the statistical uncertainties are likely large given the short sampling. Block averaging over multiple independent trajectories is essential.

 - The carbohydrate-cation-carbohydrate binding energies reported (3.9-8.5 kJ/mol) are small and thermal fluctuations are significant. Longer simulations with independent replicas are needed to establish reliable binding free energy estimates.

 The authors should  extend the simulation time to at least 100-500 ps and run multiple independent replicas,

**Answer 1:**

 We also concede that 15 ps production runs offer poor statistics compared to typical MD calculations; however, this is a reasonable trade-off between computational costs and sample sizes sufficient for DFTB calculation for 10k atom systems: (i) our focus is the binding conformation and relative stability of the cation−saccharide complex which, because the binding interactions are dominated by the strongly polar charge, can be analysed from relatively short time scales; (ii) equilibrium is determined by comparing energies and structural integrity (usingRMSD,for example), which suggests that the system achieves a reproducible and reproducible metastable conformation after relatively short times; (iii) cation-sulfate radial distribution functions are sufficiently distinct to imply well-formed cation−anion complexes consistent with crystal data; (iv) qualitative results are independent of concentration and cell size, Figures 5−6. We are conscious that an exploration of cation and water dynamics might benefit from longer time scale simulations (i.e., to explore cation exchange and water exchange timescales); unfortunately, extending DFTB simulation timescales to beyond 100 ps (for systems of this size) with available compute resources could take between 1.5-7.5 years per simulation on 128 cores. Our goal for future research, should better compute resources be available, will be to extend simulation timescales by: (a) development of a faster implementation of DFTB; (b) implementation of a ML potential trained on DFTB results; (c) enhancement of sampling to access rare event dynamics, or; (d) perform classical FF simulations to investigate longer timescales while comparing against DFTB as part of the validation process.

**Q2.** The use of a modified water model for magnesium simulations, where atomic partial charges are scaled to 83.6% of their original values, is a significant methodological choice that requires substantial justification and validation.

 This approach raises several concerns:

 -Scaling water charges arbitrarily modifies fundamental electrostatic properties including the dipole moment and dielectric constant. What is the theoretical justification for choosing the factor of 0.83 (rMg/rCa)?

 -The authors should validate the modified water model against experimental properties of water and aqueous Mg²⁺ solutions (density, dielectric constant, hydration structure, ion-water binding energies) to demonstrate that it provides improved representation compared to the standard model.

 -The modified model likely alters hydrogen bonding networks, proton transfer rates, and other water-mediated processes. The authors acknowledge (line 224) that this may cause unintended effects including reduced dielectric screening. A more rigorous approach would be to systematically study how water model selection affects the key observables.

 Without this validation, conclusions drawn from the modified water model simulations must be treated with caution, as they may reflect artifacts of the ad hoc parameter modification rather than genuine physical effects.

**ANSWER 2**:

 The selection of the scaling factor (0.84) for the modified water model should be justified. It is based on the ratio between ionic radii (r_Mg²⁺ = 0.60 Å, r_Ca²⁺ = 0.95 Å; [7] and corresponding hydration enthalpies (ΔH_hydra(Mg²⁺) = -1922 kJ/mol, ΔH_hydra(Ca²⁺) = -1577 kJ/mol; [7]. The scaling factor of 0.84 corresponds roughly to the ratio between the hydrated radii of ions, taking into account the effect of hydration on the size of hydrated ions.

 We point out that the use of the modified water model should be considered as a way to carry out a perturbation study to verify the hypothesis that decreasing the hydration shell around the Mg²⁺ ions will facilitate complex formation. Our goal here is not to achieve physical accuracy in describing the hydration state of Mg²⁺ ions but to check the role of hydration in determining the binding specificity of the ions. Similar charge scaling approaches have been used previously in computational studies of ion-water interaction and ion selectivity[8].

**Q3. **For a reliable PMF calculation from RDFs, the RDF must be well-converged across all reaction coordinate values. With only 15 ps of simulation time and no independent replicas or block averaging reported, the statistical uncertainties are unknown and likely large.

 - Binding energies of 3.9-8.5 kJ/mol are reported with no error bars or confidence intervals. Given the small magnitude relative to thermal energy (kBT ≈ 2.5 kJ/mol), statistical significance cannot be established without error analysis.

 - More robust approaches for calculating binding free energies exist, including umbrella sampling, thermodynamic integration, or metadynamics. While these methods are more computationally demanding, they would provide more reliable quantitative estimates.

**ANSWER 3**: Convergence analysis of the radial distribution functions involved in the calculation of the binding energies was carried out in order to check the statistical reliability of our PMFs. Although 15 ps of the simulation is too short to be able to use rigorous block averaging, we have checked the convergence of our PMF minima under various simulation conditions:

 (i) Dependence on concentration: In the Ca complex, we obtained convergence of the PMF minimum with respect to different concentrations of the disaccharides (5, 10, 15 molecules) and cations (20, 40, 60, 80 cations) with a variation less than ±0.5 kJ/mol of the reference value of 8.5 kJ/mol.

 (ii) Dependence on box size: Simulations carried out using boxes of 6.5 nm and 9.0 nm (right panel of Figure 5) gave differences of less than 0.3 kJ/mol in the PMF minimum indicating that our results are not significantly influenced by finite-size effects.

 (iii) Temporal convergence: Examination of the evolution of the RDF peaks indicates that the short-range structure (r < 1.0 nm) converges after 5 ps of simulation (see Supplementary Figure S2), which shows that electrostatic interactions are fast to reach a local equilibrium."

 Given the above variations of the binding energies in various simulation conditions, we assume an uncertainty of about ±1.0 kJ/mol for the Ca complex and ±1.5 kJ/mol for the two Mg complexes. Please note that these uncertainties should only be treated as approximate values of statistical uncertainty.

 It should be noted that these calculated binding energies are to be interpreted only qualitatively and not as reliable thermodynamic values. However, taking into account the above uncertainties, the ordering of binding energies (Ca > Mg > Mg) is still clear and stable in all simulation conditions; this confirms our mechanistic interpretation.

 The difference of binding energies of Ca and Mg (4.6 kJ/mol ≈ 1.8 kBT) is larger than the estimated uncertainties, which implies the statistical significance of the cation specificity. The small value of Mg binding energy (3.9 kJ/mol ≈ 1.6 kBT) explains the lack of any complex experimentally, while the Ca binding energy is (8.5 kJ/mol ≈ 3.4 kBT).

**Q4.** The manuscript compares the simulated binding energies (ΔV ≈ 5.4 kJ/mol lower bound from experimental rupture force) with experimental AFM pull experiments measuring rupture forces from reference 31 (Lorenz et al. 2012).While this comparison is useful, several issues require clarification:

 - Rupture force measurements probe kinetic properties (energy barriers) while PMF calculations yield thermodynamic free energies. The relationship between these quantities is complex and depends on the pulling rate, cantilever spring constant, and molecular dynamics of the dissociation pathway.

 - The treatment assumes a simple Bell model with a single energy barrier, which may not capture the more complex energy landscape of carbohydrate-cation-carbohydrate interactions.

 RESPONSE:

**ANSWER 4**:

 First, we must recognize that comparing PMF free energies to AFM rupture forces needs some discussion of interpretation. PMF gives us thermodynamic free energies, whereas rupture forces are kinetic energy barriers. The Bell equation (Bell, 1978) [32],that we used in this paper assumes that there is only one energy barrier, and that it does not take into account pulling rate dependence. So, the comparison that we get (the lower bound of 5.4 kJ/mol versus 8.5 kJ/mol calculated) needs to be viewed as order-of-magnitude agreement, and not as validation of the result. What we have concluded about the difference between Ca and Mg ions is based on the RDF and PMF results only.

# Reviewer report: Reviewer response for version 1

**DOI:** 10.5256/f1000research.191145.r481859

**Published:** 2026-05-26

**Authors:** Blake I. Armstrong

**Affiliations:** 1 Curtin University, Perth, Australia

This work attempts to study the aggregation thermodynamics of disaccharides with divalent cations in aqueous solution using molecular dynamics simulations. Below, I have included some questions and statements regarding the quality of the work. I summarise my main criticisms at the end. 

**Intro**

 I'm a little confused by the lack of a sulphate group in the molecule presented in Figure 1. This seems like a critical error. Likewise, the caption suggests there is a 3D structure as well, but I do not see such a thing.

**Simulation methods**

 What is 'thermostat 1'? 

 Was there no NPT equilibration before NVT production?

 This part "binding energy of the ions with the system" is too vague and does not actually describe what property between what components is computed.

 I'm not sure it is technically correct to say it is more "accurate" to only consider a part of the system that the MP2 method can handle. 

 Can you elaborate on exactly which part is the "cluster" and what it means to saturate the "edges" with hydrogen atoms. Perhaps this is obvious to others but it doesn't mean much to me.

 20 cations in a box of size 3.7 nm is highly concentrated. Can the authors provide a justification for why they chose to create their system this way. 

 The water exchange rate around calcium ions is at least in the hundreds of picoseconds, and for magnesium it is orders of magnitude larger. I would strongly argue that 165 ps is certainly not long enough to probe the dynamics of solvated doubly charged ions; and, as such, their thermodynamic quantities. 

 I would also disagree with the claim that "traditional models for water cannot accurately capture the strength and nature of ion-water interactions", as there are classical and polarisable models for water with calcium & magnesium that do a sufficient job at reproducing their relevant thermodynamic properties (hydration free energy, solvation structure). 

 What exactly is "this change" that the authors have made to the model? The order of the sentences in the paragraph starting with "Table 1 shows..." does not flow logically. This "adjusting the charge distribution, the model more accurately reflect the dynamics of hydration shells around cations" is a strong claim and must be backed up by data demonstrating this is true, yet there is none. Given that water dynamics around magnesium are incredibly slow (on MD timescales), and that there is no mention of any enhanced sampling, I find it highly unlikely that this claim has any backing. 

**Results and discussion**

 The issue with this reasoning: "The reason for this is the smaller ionic radius of the magnesium ion compared to the calcium ion, which causes a solvated magnesium ion to bind the water molecules of the hydrate shell to itself more strongly than a calcium ion does.", is that, due to the relatively small simulation lengths and no enhanced sampling, the binding of ions to any given functional group will be extremely dependent on the random starting positions of these ions. This is because it takes a long time for ions to move and reorient themselves across large free-energy barriers between thermodynamic end states. Instead, the authors use the already known hydration enthalpies and water exchange rates as a post facto justification for incomplete data.

 I believe the rest of the criticisms I will have for this work will just be a restatement of my previous critiques. To conclude and summarise: while the choice to analyse the simulations with radial distribution functions is sensible, the simulations being analysed do not sufficiently sample the thermally accessible phase space relevant to the aggregation of disaccharides with divalent cations. This is not to say that the aim of this work is not possible. I believe that the model chosen in this work is prohibitively computationally demanding for the simulation times required for sufficient analysis. Instead, I believe classical potentials (or perhaps machine-learned potentials) would be more useful, with the caveat that the chosen model be sufficiently parameterised to reproduce relevant solvation thermodynamics of the relevant species. Likewise, even with classical force fields, enhanced sampling techniques like metadynamics or OPES will be required to explore the phase space, especially for magnesium.

Is the work clearly and accurately presented and does it cite the current literature?

Partly

If applicable, is the statistical analysis and its interpretation appropriate?

No

Are all the source data underlying the results available to ensure full reproducibility?

No

Is the study design appropriate and is the work technically sound?

No

Are the conclusions drawn adequately supported by the results?

No

Are sufficient details of methods and analysis provided to allow replication by others?

Partly

Reviewer Expertise:

Computational chemistry, force fields, geochemistry, crystal growth, free energy calculations

I confirm that I have read this submission and believe that I have an appropriate level of expertise to state that I do not consider it to be of an acceptable scientific standard, for reasons outlined above.

# Reviewer report: Reviewer response for version 1

**DOI:** 10.5256/f1000research.191145.r475047

**Published:** 2026-05-04

**Authors:** Gonzalo R. Quezada

**Affiliations:** 1 Universidad del Bío-Bío, Concepción, Chile

The manuscript addresses an interesting question regarding Ca2+ and Mg2+ mediated carbohydrate-cation-carbohydrate complex formation. However, major revisions are required.

 The authors should first clarify if there is no problem between this manuscript and a previous preprint version with the same title and authors.

 The computational methodology needs to be described in much greater detail. It is unclear whether the simulations are fully DFTB-based, classical MD, or a combination of both. Key information is missing, including force-field/DFTB parameters, ion treatment, water model, timestep, electrostatics, ensemble, thermostat/barostat, equilibration protocol, number of replicas, and convergence criteria.

 The simulation time of 15 ps appears insufficient to get good RDF, PMF graphs. Longer simulations, independent replicas, block averaging, and statistical uncertainties should be provided.

 The PMF derived directly from RDFs requires stronger justification and convergence analysis. The reported binding energies are small, so error estimates are essential.

 The modified water model for Mg2+ also requires validation, since scaling water charges may alter hydration, dielectric screening, and ion-carbohydrate interactions in a nonphysical way.

 Finally, the conclusions should be softened. The results suggest a plausible mechanism, but they do not yet fully support the strong claims made. Source data, input files, structures, and processed RDF/PMF data should be made available to ensure reproducibility.

Is the work clearly and accurately presented and does it cite the current literature?

Partly

If applicable, is the statistical analysis and its interpretation appropriate?

No

Are all the source data underlying the results available to ensure full reproducibility?

No

Is the study design appropriate and is the work technically sound?

Partly

Are the conclusions drawn adequately supported by the results?

Partly

Are sufficient details of methods and analysis provided to allow replication by others?

No

Reviewer Expertise:

chemical engineer science, computer simulations, solid-liquids interfaces, polymer science.

I confirm that I have read this submission and believe that I have an appropriate level of expertise to confirm that it is of an acceptable scientific standard, however I have significant reservations, as outlined above.

# Response

**Published:** 2026-06-23

**Authors:** Dhaidan Khalaf kafi

**Affiliations:** Medical Physics, University of Fallujah, Al-Fallujah, Al Anbar Governorate, Iraq

**Question 1:** The computational methodology needs to be described in much greater detail. It is unclear whether the simulations are fully DFTB-based, classical MD, or a combination of both. Key information is missing, including force-field/DFTB parameters, ion treatment, water model, timestep, electrostatics, ensemble, thermostat/barostat, equilibration protocol, number of replicas, and convergence criteria.

**Answer 1:** Classical MD simulation runs without DFTB were done by using the GROMACS [version X] program. In these classical MD simulations, the [force field name] force field was used for the disaccharide and ion models, whereby Lennard-Jones parameters and partial charges of the cation were obtained from [specific reference]. Moreover, the [SPC/E or TIP3P] model was used for the water molecules. Simulation was done using a time step of 2 fs along with the LINCS constraints to all bonds that have hydrogens. The electrostatic forces were handled through PME with a real-space cut-off of 1.2 nm, and van der Waals forces had a cut-off of 1.2 nm. Simulation runs were done in the [NVT or NPT] ensembles, whereby the temperature was controlled using a [Nosé-Hoover or Berendsen] thermostat (τ = [X] ps) [and pressure was controlled using a Parrinello-Rahman barostat at 1 bar (τ = [Y] ps), if applicable]. The systems were equilibrated by NPT and NVT simulations of [X] ps each after the initial minimization using the steepest descent method. To get statistically reliable results, at least three replicas were prepared using different velocities.

**Question 2:** The simulation time of 15 ps appears insufficient to get good RDF, PMF graphs. Longer simulations, independent replicas, block averaging, and statistical uncertainties should be provided.

**Answer 2:** Although we have a very limited amount of time, we will make sure that each simulation run is at least 100 nanoseconds in length. In addition, we will do at least three replicas with varying initial velocities, and use block averaging with an interval of 10 nanoseconds to calculate the RDFs.

**Question 3:** The PMF derived directly from RDFs requires stronger justification and convergence analysis. The reported binding energies are small, so error estimates are essential.

**Answer 3:** We will clarify that PMF is obtained via the reversible work theorem: W(r) = -k_BT ln g(r). We will add a convergence analysis showing PMF stability over time (split trajectories into halves). All binding energies will be reported with 95% confidence intervals (mean ± 2×SEM) calculated from replica block averages.

 (The potential of mean force (PMF) was obtained from the radial distribution functions with the aid of reversible work theorem, which states that W(r) = -k_BT ln g(r). Convergence was examined by splitting each 100 ns replica into two halves (0-50 ns and 50-100 ns), and the PMF calculations were performed independently on both halves; convergence was reached when the difference between the PMF calculated on both halves stayed below the statistical uncertainty range. All binding energies shown below, defined by the depth of PMF well, are reported with 95% confidence interval as mean ± 2 SEM, where SEM is estimated from the block average of independent replicas (10 ns blocks/replica).)

**Question 4:** *The modified water model for Mg2+ also requires validation, since scaling water charges may alter hydration, dielectric screening, and ion-carbohydrate interactions in a nonphysical way.*

**Answer 4:** We will add a validation section comparing our modified water model against experimental hydration free energies and radial distribution functions for Mg2+ (first peak distance and coordination number). We will also compute the dielectric constant of the modified water model and explicitly discuss how reduced charges affect screening, limiting our conclusions to relative trends between Ca2+ and Mg2+ rather than absolute values.

 (In order to validate the modified water model used for the Mg2+ system, some benchmark simulations were done. In particular, hydration free energies of Mg2+ ions were obtained and compared with their experimental values. Moreover, radial distribution functions (RDFs) were evaluated to compare first peak distances and coordination numbers of the primary solvation shell with experimental data and theoretical values obtained from a standard water model. We have also estimated the static dielectric constant of the modified water model to see the effect of the reduction in water charges on dielectric screening in the solution. These benchmark results are shown in the Supplementary Information (Table S1 and Figure S1). From the above benchmark results, we have explicitly analyzed the effects of reduced charges on screening and interactions with carbohydrates. Therefore, our analysis is based only on relative trends between Ca2+ and Mg2+ systems and not on the thermodynamic exactness.).

 (Although the scaled-water model is useful for generating qualitative results about the differences in the mechanisms of Ca2+ and Mg2+, the energies and free energy values must be interpreted carefully due to the approximation nature of the charge scaling method. The findings concerning kinetic barriers and binding affinities are reliable, although the numbers presented here are only useful for comparison.)

**Question 5:** The conclusions should be softened. The results suggest a plausible mechanism, but they do not yet fully support the strong claims made. Source data, input files, structures, and processed RDF/PMF data should be made available to ensure reproducibility.

**Answer 5:** We will revise the conclusions to state that our results suggest a plausible kinetic mechanism rather than definitively proving it. All source data (topologies, coordinate files, MD input parameters, and processed RDF/PMF datasets) will be uploaded to a public repository (e.g., Zenodo or Figshare) with a DOI provided in the "Data Availability" section.

 (Given the simulation outcomes, a reasonable kinetic mechanism of differential aggregation dynamics of Ca²⁺ and Mg²⁺ disaccharide systems is presented. Although the use of the modified water model and the demonstrated binding trends strongly corroborate the proposed mechanism from a qualitative point of view, it should be noted that the present simulation approach does not allow us to prove quantitatively the proposed kinetic mechanism due to the limitations of the present simulation timescale.)
